# Intracranial Arterial Dolichoectasia in Spontaneous Intracerebral Hemorrhage: A Retrospective Cross-Sectional Study of Morphometric Features and Cerebral Small Vessel Disease

**DOI:** 10.3390/jcm15176881

**Published:** 2026-09-05

**Authors:** Nazakat Nurmamat, Yunfang Luo, Weijia Xie, Xianjing Feng, Fang Yu, Yinghuan Pan, Hesham A. Alyamani, Yang Du, Jian Xia

**Affiliations:** 1 Department of Neurology, Xiangya Hospital, Central South University, 87# Xiangya Road, Changsha 410008, China; 238102094@csu.edu.cn (N.N.);; 2 Clinical Research Center for Cerebrovascular Disease of Hunan Province, Central South University, Changsha 410008, China; 3 National Clinical Research Center for Geriatric Disease, Xiangya Hospital, Central South University, Changsha 410008, China

**Keywords:** intracranial arterial dolichoectasia, intracerebral hemorrhage, cerebral small vessel disease, tortuosity

## Abstract

**Objectives:** The aim of this study was to determine the prevalence and distribution of intracranial arterial dolichoectasia (IADE) across the anterior and posterior circulations and assess its association with cerebral small vessel disease (CSVD) imaging markers in patients with spontaneous intracerebral hemorrhage (ICH). **Methods:** This retrospective cross-sectional study included 174 ICH patients. Three-dimensional vascular reconstruction was used to assess cavernous internal carotid artery morphology, vertebrobasilar dolichoectasia, and exploratory middle cerebral artery (MCA) tortuosity. IADE was defined by Type III–IV cavernous internal carotid artery morphology and/or vertebrobasilar dolichoectasia. Multivariable logistic regression was adjusted for age, sex, hypertension, diabetes mellitus, and previous stroke. **Results:** IADE was present in 52 patients (29.9%) and was observed more frequently in CSVD-related ICH than in macrovascular ICH (32.9% versus 14.3%; *p* = 0.049). Among patients with CSVD-related ICH, IADE was associated with deep cerebral microbleeds (adjusted odds ratio [OR], 3.42; 95% CI, 1.27–9.20), severe basal ganglia enlarged perivascular spaces (adjusted OR, 4.24; 95% CI, 1.72–10.46), and deep white matter hyperintensity grade ≥ 2 (adjusted OR, 3.71; 95% CI, 1.59–8.65). These associations were still significant after false discovery rate correction. No significant association was observed with lobar cerebral microbleeds. Exploratory analyses showed higher maximum and mean bilateral MCA tortuosity indices in patients with IADE. **Conclusions**: IADE was present in approximately one third of patients with ICH and was observed more frequently in CSVD-related than in macrovascular ICH in this cohort. Its association with deep CSVD imaging markers suggests a possible link between IADE and deep small-vessel disease, while the MCA findings remain exploratory.

## 1. Introduction

Intracranial arterial dolichoectasia (IADE) is a disorder in which the major cerebral arteries show pathological dilation, elongation, or increased curvature. It is increasingly recognized on neuroimaging in patients with cerebrovascular disease [1,2]. Its diagnosis still relies largely on visual assessment of vascular morphology. The lack of standardized diagnostic criteria has contributed to substantial variation in reported prevalence, ranging from 2.6% to 17.1% among stroke populations [3,4,5]. The Smoker criteria are widely used to define vertebrobasilar dolichoectasia (VBD) [6], which has led most studies to focus on posterior circulation involvement and its association with ischemic stroke [7]. However, comprehensive assessments across multiple intracranial arteries remain limited. The prevalence, morphological patterns, and clinical relevance of IADE in patients with intracerebral hemorrhage (ICH) are also not fully understood.

Cerebral small vessel disease (CSVD) is thought to underlie approximately 80% of spontaneous ICH cases. CSVD involves structural changes in small penetrating arteries and arterioles and is an important substrate for spontaneous ICH, particularly in deep brain regions [8]. In contrast, macrovascular and structural causes, including arteriovenous malformations (AVMs), dural arteriovenous fistulas (dAVFs), or cerebral cavernous malformations (CCMs), account for 15–23% [9]. A recent study of chronic subdural hematoma highlighted vascular remodeling and fragility in hemorrhagic pathology [10]. Pathological studies have also shown that dolichoectatic arteries exhibit arterial wall remodeling, including increased external diameter and wall thinning. These structural abnormalities have been proposed to alter local hemodynamics and pulsatile stress in the distal circulation [1]. In clinical studies, patients with IADE have also shown a higher burden of CSVD markers, including lacunes, white matter hyperintensities (WMH), cerebral microbleeds (CMBs), and enlarged perivascular spaces (EPVS) [11,12,13,14]. Studies on VBD have also suggested that cerebral microbleeds may preferentially involve deep or vertebrobasilar territories [7]. These findings suggest a possible link between large-artery morphological abnormalities and small-vessel injury, although the nature and direction of this relationship remain uncertain. However, it remains unclear whether IADE is preferentially associated with CSVD-related ICH and deep small-vessel injury or reflects a more general intracranial arterial phenotype across different ICH etiologies.

We therefore hypothesized that IADE would be associated with CSVD-related imaging abnormalities and might differ across ICH etiologies. We analyzed 174 patients with spontaneous ICH using three-dimensional vascular reconstruction to characterize cavernous internal carotid artery (cICA) and vertebrobasilar morphology. Associations of IADE with ICH etiology, hemorrhage location, and established CSVD imaging markers were examined. We also explored the morphology and tortuosity index of the middle cerebral artery, with a particular focus on the M1 segment, which supplies brain regions commonly affected by deep hypertensive hemorrhage. Together, these analyses aimed to characterize the vascular and neuroimaging features associated with IADE in patients with spontaneous ICH.

## 2. Patients and Methods

This study was a retrospective cross-sectional analysis of prospectively collected data from a single-center cohort of patients with spontaneous, nontraumatic intracerebral hemorrhage. The cohort was established prospectively after ethics approval, with consecutive enrollment and prospective collection of clinical and imaging data. The study protocol was approved by the Medical Ethics Committee of Xiangya Hospital, Central South University (No. 202401026; 25 January 2024), with continuing approval (No. 2026061315; valid until 1 February 2027). All participants provided written informed consent at enrollment.

### 2.1. Participants

Consecutive patients diagnosed with spontaneous, nontraumatic ICH who were admitted to Xiangya Hospital, Central South University, between February 2024 and December 2025 were enrolled in the study. All participants were recruited within 30 days after the onset of symptoms. The 30-day window was defined by the underlying ICH cohort protocol to capture patients during the acute and subacute phases while allowing completion of standardized clinical and neuroimaging assessments.

**Figure 1:**
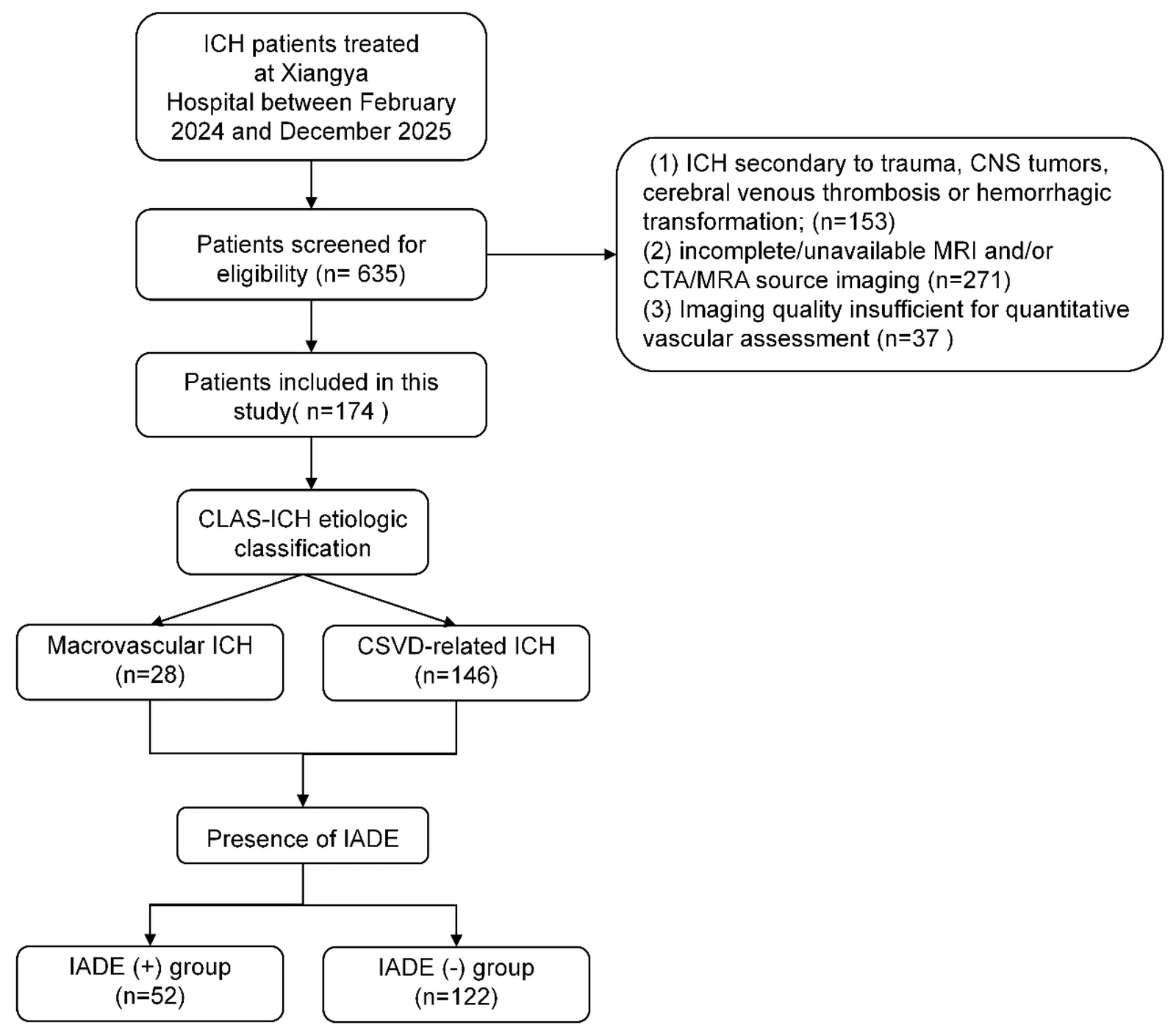
Participant Enrollment Flowchart.

Patients meeting any of the following conditions were excluded: (1) ICH secondary to trauma, central nervous system tumors, cerebral venous thrombosis, or hemorrhagic transformation; (2) incomplete or unavailable brain MRI or CTA/TOF-MRA source images suitable for standardized analysis; or (3) inability to reliably assess the arterial segments required for primary IADE classification. The first group was excluded because these conditions represent hemorrhagic mechanisms distinct from the spontaneous ICH etiologies examined in this study. Complete brain MRI was required for standardized assessment of CSVD markers, whereas evaluable CTA or TOF-MRA was required for three-dimensional vascular reconstruction and morphometric assessment. Figure 1 presents the participant enrollment process.

To establish etiology, a standardized diagnostic workup, including neurological examination, laboratory tests, and relevant neuroimaging, was performed in all patients. Etiologic classification was based on the Causal Classification System for Intracerebral Hemorrhage Subtypes (CLAS-ICH) [9]. ICH subtypes attributed to arteriolosclerosis, cerebral amyloid angiopathy, mixed CSVD, and other rare forms of CSVD were categorized as CSVD-related ICH. Patients with identified structural vascular lesions (e.g., vascular malformations, dural arteriovenous fistulas, moyamoya, or cerebral cavernous malformations) were categorized as the macrovascular ICH.

### 2.2. Imaging Protocol

Brain MRI was acquired at either 1.5 or 3.0 T. The protocol comprised T1- and T2-weighted sequences, FLAIR, and SWI. Intracranial vascular imaging was obtained with either CTA or three-dimensional TOF-MRA. Of the 174 patients, 34 were examined by CTA and 140 by TOF-MRA. The same vascular reconstruction procedure and anatomical landmarks were applied to CTA and TOF-MRA images. Hematoma volume was estimated using the ABC/2 method [15]. Hematoma location was classified as lobar, deep, or infratentorial, or uncertain according to the anatomical distribution of the hemorrhage.

#### 2.2.1. Assessment of CSVD Imaging Markers

Assessment of CSVD imaging markers followed the STRIVE-2 recommendations [14,16]. Lacunes and cerebral microbleeds (CMBs) were categorized by anatomical location [17]. CMBs were recorded as deep, lobar or infratentorial, and both their presence and number were recorded. Periventricular WMH was graded from 0 to 3, and grade 3 was considered severe. Deep WMH was also graded from 0 to 3, and grade ≥ 2 was used to indicate a moderate-to-severe burden in the primary analysis [18]. Enlarged perivascular spaces (EPVS) were graded separately in the basal ganglia (BG) and centrum semiovale (CSO) on axial T2-weighted images using a validated five-point scale, from 0 (none) to 4 (>40) [16]. A grade of 3–4 was defined as severe EPVS [19]. CSVD burden was quantified using a validated global score (range 0–6). Etiology-specific burdens were further assessed with an HTNA-CSVD score (range 0–4) and a CAA-CSVD score (range 0–6), consistent with established methodology [20]. Probable or possible CAA was identified according to the Boston criteria version 2.0 [21,22]. W.X. and Y.P. independently evaluated all CSVD markers while blinded to vascular morphology and the clinical information.

#### 2.2.2. Assessment of IADE

Three-dimensional vascular reconstructions were generated from CTA or TOF-MRA images using Mimics Research software, version 21.0. The reconstructed vessels included the basilar artery (BA), bilateral intracranial internal carotid arteries (ICAs), and bilateral M1 segments of the middle cerebral arteries (MCAs). For each arterial segment, the vessel centerline, centerline length, end-to-end straight-line distance, maximum luminal diameter, and morphological features were assessed. The Tortuosity Index (TI) was defined as (centerline length/straight line distance) × 100 [23].

The BA was evaluated using the Smoker criteria [24]. Maximum BA diameter, laterality score, and bifurcation-height score were recorded. Vertebrobasilar dolichoectasia (VBD) was defined as a BA diameter > 4.5 mm together with a laterality score ≥ 2 and/or a bifurcation-height score ≥ 2 [25]. Tortuosity Index was also calculated for the entire BA.

**Figure 2:**
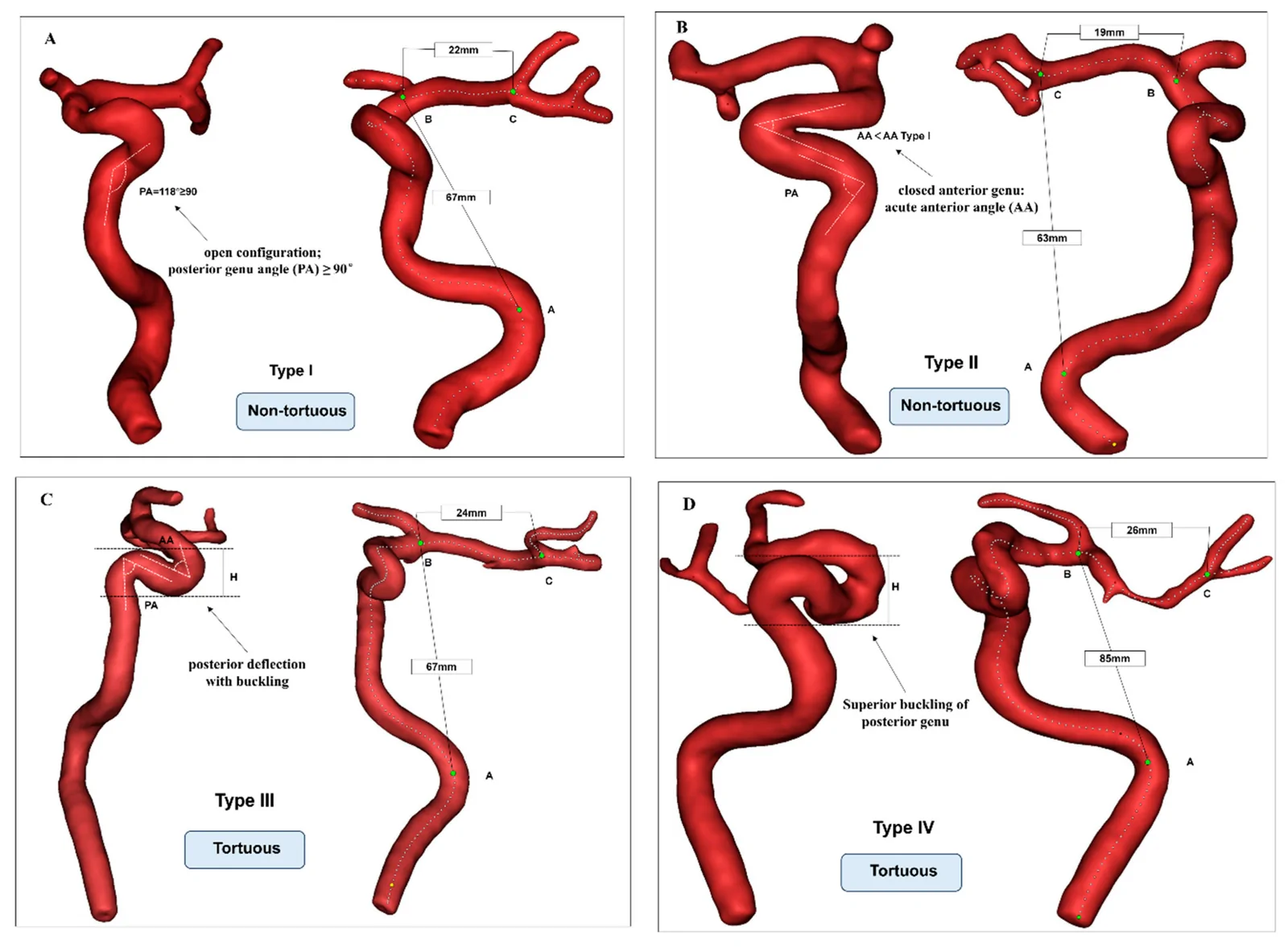
Classification of cavernous internal carotid artery (ICA) tortuosity and methodology for intracranial ICA and (Middle cerebral artery) M1 segment length measurements. Four patients are arranged in a 2 × 2 grid, each presenting paired images: cavernous segment morphology (**left**) and full-length intracranial ICA with centerline (right). Cavernous ICA tortuosity was classified into four types based on anterior/posterior genu geometry [26,27]: (**A**) Type I—open configuration with posterior genu angle (PA) ≥ 90°; (**B**) Type II—closed anterior genu with more acute anterior angle (AA) than Type I; (**C**) Type III—posterior deflection with buckling; (**D**) Type IV—most tortuous, Simmons-catheter morphology (posterior genu buckled superiorly; height difference H indicated). Types III and IV were considered tortuous. On each full-length view, anatomical landmarks are marked: A, petrous genu; B, terminal ICA bifurcation; C, first major MCA bifurcation or trifurcation. Intracranial ICA (A–B) and M1 (B–C) lengths were measured along the centerline. The corresponding straight-line distance was measured directly between the same endpoints, (values in mm). M1 morphology was assessable: straight (Type I, III), single-curve (Types II, IV).

Cavernous internal carotid artery (cICA) tortuosity was categorized using an established geometric classification system [26,27]. Each ICA was assigned to one of four types according to the morphology of the anterior and posterior genu: Type I (Open configuration): characterized by a posterior angle (PA) ≥ 90°. Type II (Closed anterior genu): defined by a more acute anterior angle (AA) than Type I. Type III (Posterior deflection): exhibits posterior deflection with buckling. Type IV (Severe tortuosity): characterized by a Simmons-catheter morphology in which posterior genu buckles superiorly to the anterior genu. A cavernous ICA exhibiting Type III or IV geometry was categorized as tortuous (Figure 2). In addition to the segment-specific geometric classification, the overall tortuosity of the entire intracranial ICA segment was evaluated as a complementary objective metric. This segment was defined from the petrous genu (anterior genu of the petrous segment) to the terminal bifurcation into the middle and anterior cerebral arteries [23]. TI was also calculated for this segment. This continuous variable provides a quantitative measure of global tortuosity beyond the cavernous segment, capturing elongation and complexity throughout the vessel’s full intracranial course. To evaluate possible diameter-defined cICA ectasia, the maximum luminal diameter of each cavernous ICA was also measured. A threshold of ≥7.0 mm was used in a post hoc sensitivity analysis. For the anterior circulation, the primary IADE definition was morphology-based and did not require concomitant cICA dilation; diameter-defined cICA ectasia was evaluated separately in a post hoc sensitivity analysis.

The MCA-M1 segment was defined from the terminal bifurcation of the internal carotid artery to the first major MCA bifurcation or trifurcation [28]. The overall MCA-M1 morphological pattern was categorized as straight, single-curve, or S-shaped. MCA-TI was calculated as (M1 centerline length/end-to-end straight-line distance) × 100 using these same landmarks. The maximum bilateral MCA-TI was defined as the higher value of the two sides, and the mean bilateral MCA-TI was calculated as their average. For morphology-based analyses, the more severe bilateral pattern was selected using the following ordered classification: straight, single-curve, and S-shaped. MCA measurements were considered exploratory and were not included in the primary IADE definition.

The primary IADE phenotype was defined as the presence of cICA tortuosity corresponding to Type III or IV morphology on either side and/or VBD meeting the combined diameter and positional criteria. Patients meeting either criterion were classified as IADE-positive; all remaining patients were classified as IADE-negative.

Investigators assessing IADE and vascular morphology were blinded to the CSVD imaging findings and clinical information. Interobserver reproducibility was assessed in a stratified random sample of 30 patients by two independent observers blinded to each other’s assessments. Interobserver agreement was almost perfect for categorical imaging variables, with κ values ranging from 0.87 to 0.92. Continuous vascular measurements showed excellent reproducibility, with ICCs ranging from 0.92 to 0.95.

### 2.3. Statistical Analysis

Continuous variables are reported as mean (SD) or median (interquartile range), whereas categorical variables are presented as number and percentage. Between-group comparisons used Welch’s *t*-test or the Mann–Whitney U test for continuous variables and Pearson’s chi-square or Fisher’s exact test for categorical variables, as appropriate. Logistic regression models were subsequently used to examine the relationships of IADE with ICH location, etiologic subtype, and CSVD imaging markers. Multivariable models were adjusted for age, sex, hypertension, diabetes mellitus, and any previous stroke. The association between IADE status and maximum bilateral MCA tortuosity index was evaluated using multivariable linear regression with adjustment for the same covariates. We compared MCA tortuosity indices across straight, single-curve, and S-shaped morphological groups using the Kruskal–Wallis test with Dunn–Bonferroni pairwise comparisons. Differences in the distribution of bilateral MCA morphological patterns according to IADE status were assessed using the Fisher–Freeman–Halton exact test. To account for multiple comparisons, false discovery rate correction using the Benjamini–Hochberg method was applied across three prioritized deep CSVD imaging outcomes: deep CMBs, severe BG-EPVS, and deep WMH grade ≥ 2. These outcomes were prioritized because they represent complementary deep CSVD imaging domains and have previously been associated with IADE [11,12,13]. Other CSVD outcomes were considered exploratory. Phenotype-specific and subtype-stratified sensitivity analyses were performed for the three prioritized deep CSVD outcomes. Additional sensitivity models further adjusted for vascular imaging modality or smoking, dyslipidemia, and alcohol use. Because some subgroup analyses involved limited numbers of events, Firth-penalized logistic regression was additionally performed as a robustness analysis. Exploratory interaction analyses between cICA tortuosity and VBD were performed. IADE classification and variables included in the primary analyses were complete. Missing data for other variables were analyzed using available observations without imputation. Statistical significance was defined as a two-sided *p* value < 0.05. Analyses were conducted using SPSS software (version 27).

## 3. Results

### 3.1. Study Population and Prevalence of IADE

**Figure 3:**
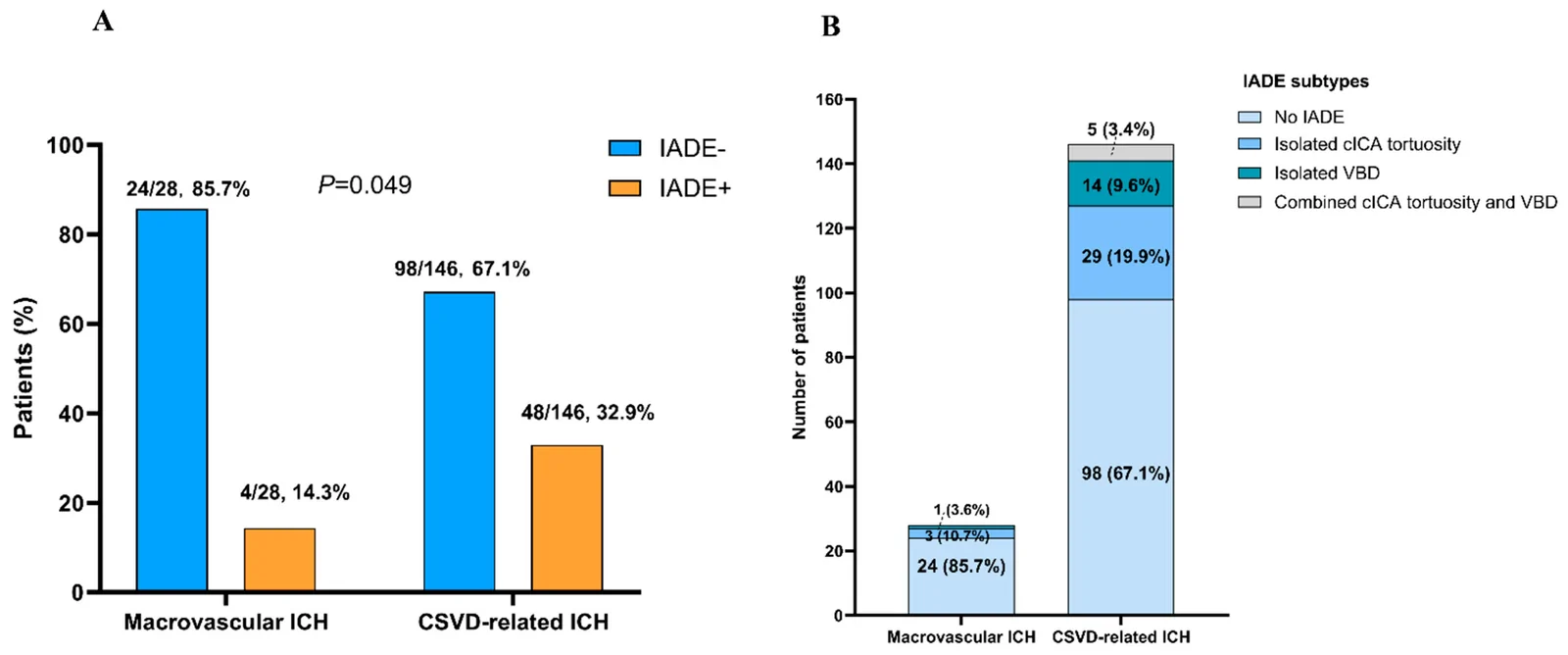
Distribution of IADE according to broad ICH etiology. (**A**) Proportions of IADE-negative and IADE-positive patients in the macrovascular ICH and CSVD-related ICH groups. IADE was present in 4 of 28 patients (14.3%) with macrovascular ICH and 48 of 146 patients (32.9%) with CSVD-related ICH (*p* = 0.049). (**B**) Absolute numbers of patients with no IADE, isolated cICA involvement, isolated VBD, or combined cICA and VBD involvement in each etiologic group. The subtype distribution was interpreted descriptively because of the small numbers in several categories.

A total of 174 patients with spontaneous, nontraumatic ICH were included. Of these, 146 (83.9%) had CSVD-related ICH and 28 (16.1%) had macrovascular ICH. IADE was identified in 52 patients (29.9%) and was observed more frequently in the CSVD-related ICH group than in the macrovascular ICH group (48/146 [32.9%] vs. 4/28 [14.3%], *p* = 0.049; Figure 3A). IADE most commonly involved the cICA alone (32/174, 18.4%), followed by isolated VBD (15/174, 8.6%) and combined cICA–VBD involvement (5/174, 2.9%). The distribution of IADE subtypes according to ICH etiology is shown descriptively in Figure 3B. The macrovascular ICH group included 14 cerebral cavernous malformations, 9 arteriovenous malformations, 3 dural arteriovenous fistulas, and 2 cases of moyamoya disease (Supplementary Table S2). The mean maximum diameters of the right and left cICAs were 5.03 ± 0.55 mm and 5.12 ± 0.54 mm, respectively. Only one patient met the literature-based threshold of ≥7.0 mm for diameter-defined cICA ectasia. Adding this single case did not materially change the IADE classification or the primary results. The morphology-based IADE definition was therefore retained.

### 3.2. Baseline Characteristics According to IADE Status

The cohort was divided into 122 IADE-negative and 52 IADE-positive patients. Table 1 summarizes the baseline characteristics of the study population. The mean age was 57.2 ± 14.7 years, and 118 patients (67.8%) were male. Patients with IADE had higher systolic blood pressure (148.6 ± 25.4 vs. 139.5 ± 25.0 mmHg, *p* = 0.030) and diastolic blood pressure (88.9 ± 14.3 vs. 83.7 ± 16.0 mmHg, *p* = 0.045). Diabetes mellitus was less common in the IADE-positive group (11.5% vs. 26.2%, *p* = 0.032). A history of stroke was also less frequent among IADE-positive patients (15.4% vs. 32.0%, *p* = 0.024), mainly because previous ischemic stroke was less common in this group (1.9% vs. 15.6%, *p* = 0.010). The frequency of previous ICH was similar between the two groups. Hematoma location was also comparable (*p* = 0.873). In the overall cohort, 57 patients (32.8%) had lobar ICH, 102 (58.6%) had deep or infratentorial ICH, and 15 (8.6%) had an uncertain location. Mixed CSVD was also more common in the IADE group (57.7% vs. 36.9%), although the overall distribution of etiologic subtypes was not statistically significant (*p* = 0.098). Hematoma volume, admission NIHSS and mRS scores, the presence of carotid plaques, and mean common carotid artery intima–media thickness did not differ significantly (Table 1).

### 3.3. Distributions of CSVD Imaging Markers

Analyses of CSVD imaging markers were restricted to the 146 patients with CSVD-related ICH. Compared with IADE-negative patients, IADE-positive patients more frequently had any CMBs (93.8% vs. 79.6%, *p* = 0.027), deep CMBs (85.4% vs. 67.3%, *p* = 0.020), severe BG-EPVS (43.8% vs. 22.4%, *p* = 0.008), and deep WMH grade ≥ 2 (72.9% vs. 52.0%, *p* = 0.016). The median BG-EPVS grade was also higher in the IADE-positive group (*p* = 0.012). Global and HTNA-CSVD scores differed between groups, whereas CAA-CSVD scores were comparable. Distributions of lacunes, lobar CMBs, infratentorial CMBs, CSO-EPVS, and periventricular WMH were comparable between the two groups (Table 2).

### 3.4. Associations of IADE with ICH Characteristics and CSVD Imaging Markers

Among patients with CSVD-related ICH, IADE was not independently associated with arteriolosclerosis, CAA, or hematoma location. Mixed CSVD was more common in IADE-positive patients and showed a borderline association after multivariable adjustment (adjusted OR, 2.03; 95% CI, 0.94–4.38; *p* = 0.071). IADE showed no significant relationship with either lobar ICH (adjusted OR, 0.82; 95% CI, 0.36–1.86; *p* = 0.636) or deep or infratentorial ICH (adjusted OR, 1.40; 95% CI, 0.64–3.05; *p* = 0.402).

**Figure 4:**
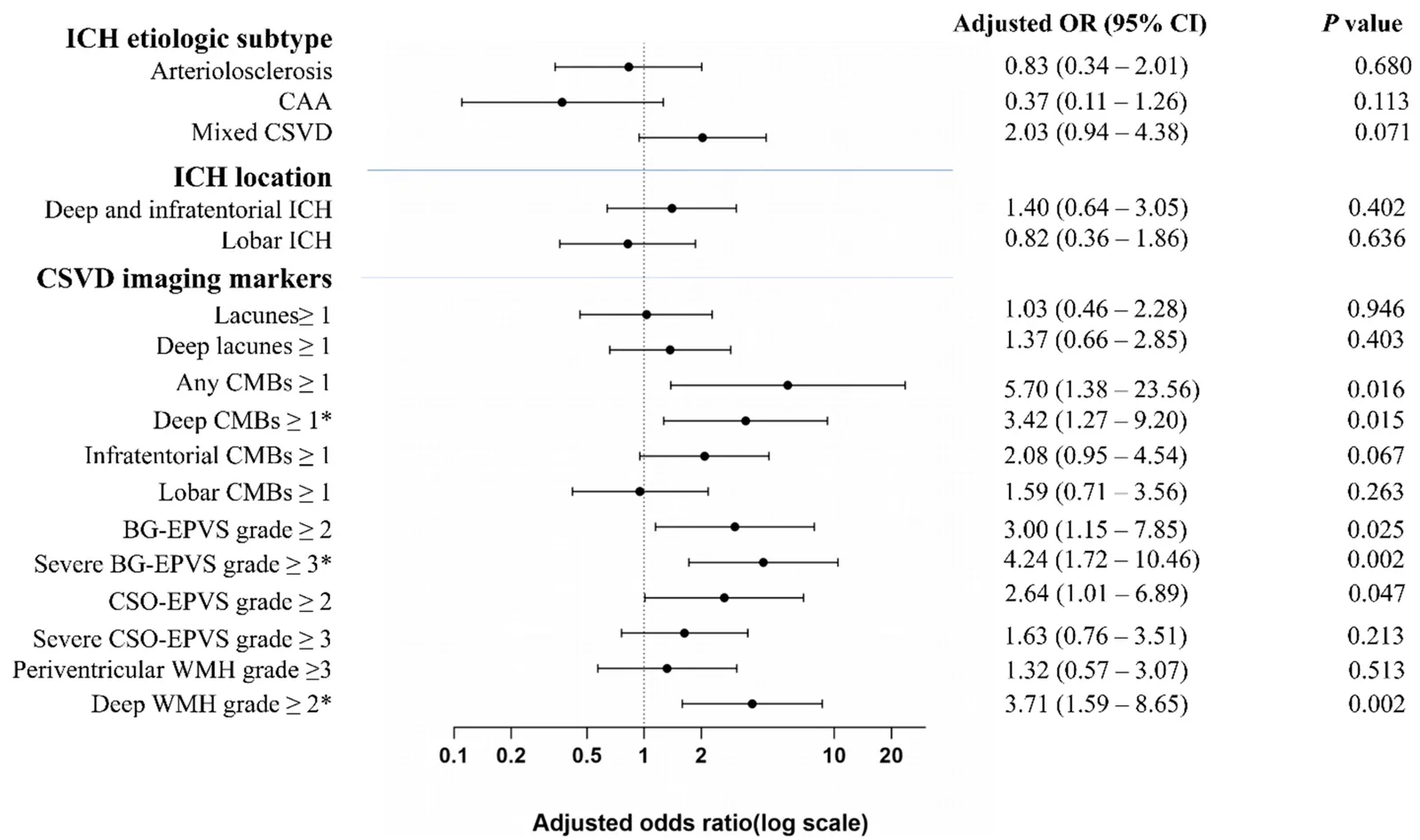
Adjusted associations of IADE with ICH characteristics and CSVD imaging markers. Analyses were restricted to patients with CSVD-related ICH (*n* = 146) and adjusted for age, sex, hypertension, diabetes mellitus, and any previous stroke. The dashed line indicates OR = 1. * Remained significant after FDR correction across the three prioritized deep CSVD outcomes.

Multivariable analyses showed significant relationships between IADE and any CMBs (adjusted OR, 5.70; 95% CI, 1.38–23.56; *p* = 0.016), deep CMBs (adjusted OR, 3.42; 95% CI, 1.27–9.20; *p* = 0.015), severe BG-EPVS (adjusted OR, 4.24; 95% CI, 1.72–10.46; *p* = 0.002) and deep WMH grade ≥ 2 (adjusted OR, 3.71; 95% CI, 1.59–8.65; *p* = 0.002). A nonsignificant trend was observed for infratentorial CMBs (adjusted OR, 2.08; 95% CI, 0.95–4.54; *p* = 0.067). In contrast, IADE showed no significant relationship with lacunes, lobar CMBs ≥ 1, severe CSO-EPVS, or periventricular WMH grade ≥ 3. After Benjamini–Hochberg false discovery rate correction across the three primary deep CSVD outcomes, the associations with deep CMBs, severe BG-EPVS, and deep WMH grade ≥ 2 remained significant, with q values of 0.015, 0.004, and 0.004, respectively (Table 3 and Figure 4).

Phenotype-specific analyses showed that the cICA tortuosity criterion was associated with severe BG-EPVS and deep WMH, whereas VBD alone was not significantly associated with the three prioritized outcomes (Supplementary Table S3). In subtype-stratified analyses, isolated cICA tortuosity was associated with all three outcomes, whereas isolated VBD was associated only with severe BG-EPVS (Supplementary Table S4). Estimates for combined cICA–VBD involvement were imprecise because of the small subgroup. No convincing evidence of interaction was observed, although estimates were limited by the small number of patients with combined cICA–VBD involvement.

Firth penalized logistic regression yielded similar estimates for deep CMBs (adjusted OR, 3.09; 95% CI, 1.26–8.53), severe BG-EPVS (adjusted OR, 3.89; 95% CI, 1.66–9.60), and deep WMH grade ≥ 2 (adjusted OR, 3.44; 95% CI, 1.55–8.09), supporting the stability of the primary findings. Additional adjustment for vascular imaging modality or smoking, dyslipidemia, and alcohol use did not materially alter the primary associations (Supplementary Table S5).

### 3.5. Quantitative Assessment of cICA Morphology

Intracranial ICA-TI was higher in arteries with Type III–IV cICA morphology than in those with Type I–II morphology on both sides (both *p* < 0.001; Supplementary Figure S1). Across Types I–IV, centerline length and intracranial ICA-TI differed significantly on both sides (all *p* < 0.001; Supplementary Table S1).

### 3.6. Exploratory Analysis of MCA Tortuosity

**Figure 5:**
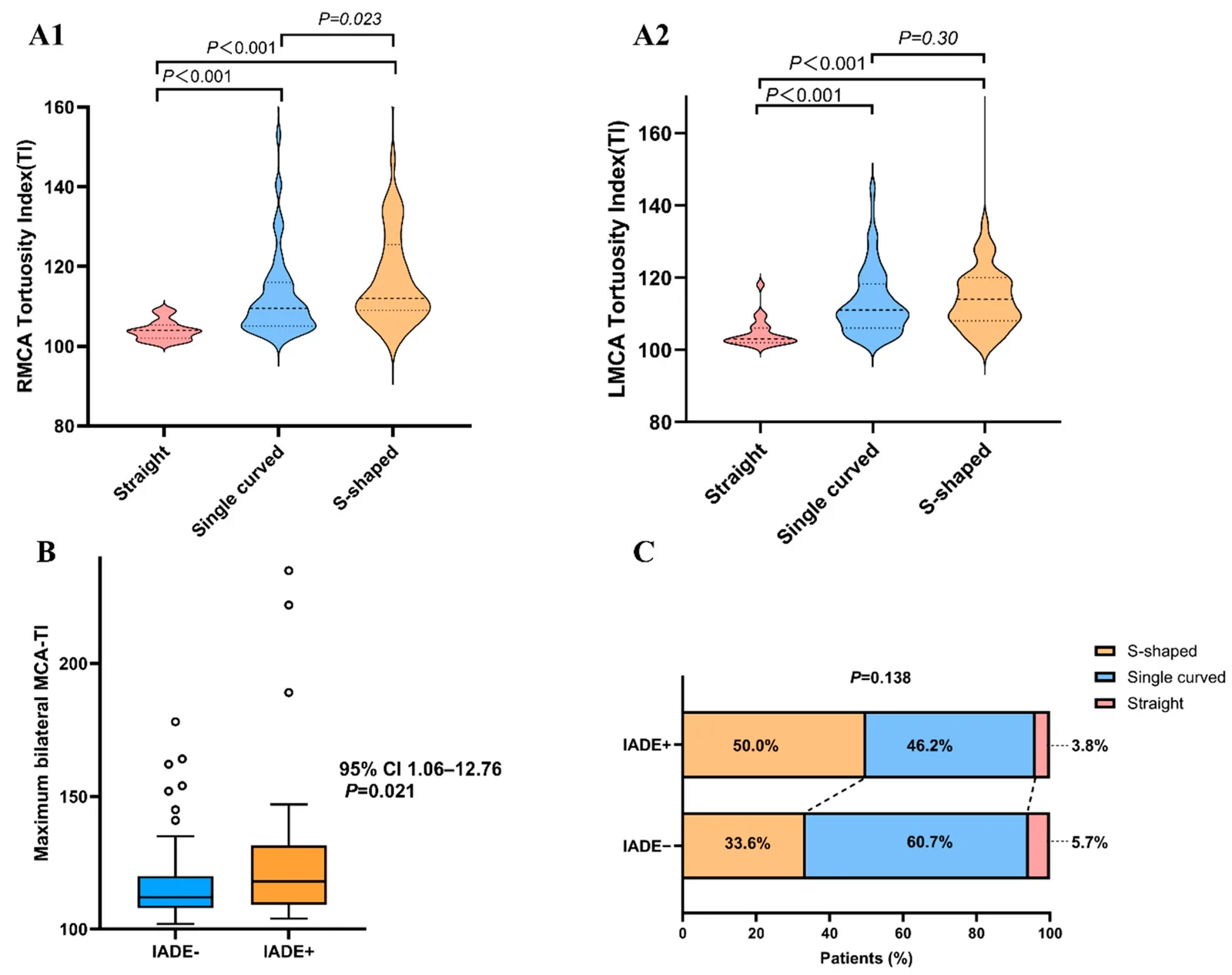
Exploratory analysis of MCA morphology and IADE. Panel (**A1**,**A2**) compares MCA tortuosity index across the three visual morphology categories—straight, single-curve, and S-shaped—for the right MCA (**A1**) and left MCA (**A2**), respectively. Overall differences were assessed using the Kruskal–Wallis test, followed by Dunn–Bonferroni post hoc pairwise comparisons. Panel (**B**) shows the distribution of the maximum bilateral MCA tortuosity index according to IADE status. Boxes indicate the median and interquartile range, whiskers represent 1.5 × IQR, and dots represent individual patients. The *p* value was derived from multivariable linear regression adjusted for age, sex, hypertension, diabetes mellitus, and previous stroke. Panel (**C**) shows the distribution of the highest-grade bilateral MCA morphology according to IADE status using a 100% stacked bar chart. For each patient, the more severe morphology between the bilateral MCA-M1 segments was selected. The overall three-category distribution was compared using the Fisher–Freeman–Halton exact test.

MCA-TI increased with greater morphological complexity in both MCAs, with higher values in single-curve and S-shaped vessels than in straight vessels (Figure 5(A1,A2); Supplementary Table S6). The maximum bilateral MCA-TI was higher in IADE-positive than in IADE-negative patients (median, 117.6 [109.6–130.3] vs. 112.4 [107.5–120.1], *p* = 0.023), and this difference remained significant after multivariable adjustment (adjusted β, 6.91; 95% CI, 1.06–12.76; *p* = 0.021; Figure 5B). Similar results were observed for mean bilateral MCA-TI (adjusted β, 5.29; 95% CI, 1.42–9.15; *p* = 0.008; Supplementary Table S7). S-shaped highest-grade bilateral morphology was more frequent in IADE-positive patients (50.0% vs. 33.6%), although the overall three-category distribution did not differ significantly by IADE status (*p* = 0.138; Figure 5C). Associations in subgroup analyses were less consistent and should be interpreted as exploratory (Supplementary Table S8).

## 4. Discussion

Among the 174 patients with spontaneous ICH, 52 (29.9%) met the criteria for IADE, with a higher proportion observed in the CSVD-related ICH group than in the macrovascular ICH group. Within the CSVD-related subgroup, IADE remained independently associated with deep CMBs, severe BG-EPVS, and deep WMH grade ≥ 2; these associations remained significant after false discovery rate correction. No significant association was observed with lobar CMBs, CAA-related measures, specific CSVD etiologic subtypes, or hematoma location. Exploratory analyses further showed greater MCA tortuosity in IADE-positive patients. Overall, these findings suggest that IADE is more closely related to deep CSVD injury and can coexist across diverse cerebrovascular pathologies.

Assessment of intracranial dolichoectasia has traditionally centered on the basilar artery, most commonly using the Smoker criteria [4,29,30]. However, this approach does not fully capture anterior circulation involvement. Although previous studies have linked vertebrobasilar dolichoectasia (VBD) to a higher risk of hemorrhagic cerebrovascular events [31,32], the distribution of comprehensive IADE across the anterior and posterior circulations remains less well characterized in patients with ICH.

IADE was identified in 29.9% of patients, with overall vertebrobasilar involvement in 11.5%. These figures are comparable to the 24% overall prevalence and 11% basilar artery involvement reported by Del Brutto et al. in ischemic stroke patients, who underwent systematic semi-automatic assessment of full main intracranial arteries [5]. However, direct comparisons should be made cautiously because of differences in study populations, imaging modalities, and diagnostic definitions. In our cohort, anterior-circulation involvement, particularly cICA tortuosity, accounted for a substantial proportion of the morphology-based IADE phenotype, whereas diameter-defined cICA ectasia was rare. Therefore, anterior-circulation involvement mainly reflected elongation and tortuosity rather than marked dilation. This distinction is relevant because cICA involvement was morphology-based, whereas VBD was defined using both diameter and positional criteria. Phenotype-specific analyses further suggested that the associations with deep CSVD were more evident for the cICA component.

In this Chinese ICH cohort, IADE was associated with deep CSVD imaging markers, particularly deep CMBs, severe BG-EPVS, and deep WMH grade ≥ 2. This finding supports and extends a growing body of evidence from diverse cohorts [1,3,7,11,12,13,33]. Similar associations have been reported in ICH cohorts [30], ischemic stroke populations [1,5,34], and younger patients with selected vascular disorders [35]. This consistency may indicate shared susceptibility of the large arteries and penetrating vessels. A recent meta-analysis also suggested that the relationship between IADE and CSVD may not be fully accounted for by conventional vascular risk factors [13]. One possible biological explanation involves abnormal extracellular matrix remodeling, including altered matrix metalloproteinase activity, which has been implicated in the development of IADE [1,12,36]. Associated medial degeneration, smooth muscle cell loss, and disruption of elastic fibers may reduce vascular elasticity and alter the transmission of pulsatile energy to distal penetrating arteries. This provides a reasonable explanation for the observed association with deep CSVD markers, although the mechanism was not directly tested in our study.

IADE was also present in 4 of 28 patients with macrovascular ICH. Given the small and heterogeneous macrovascular group, this observation is descriptive and should not be taken as evidence of a shared mechanism across ICH etiologies. The coexistence of IADE and macrovascular lesions may reflect shared arterial-wall susceptibility, hemodynamic factors, or coincidental abnormalities [37,38,39]. Genetic and extracellular matrix disorders have been associated with arterial tortuosity and dilatation [4,12,35,40,41,42,43,44,45], but genetic testing was not systematically available in this study. Further studies with larger etiologic subgroups and genetic or vessel-wall assessment are needed to determine whether IADE represents a common vascular phenotype across different causes of ICH.

In our exploratory analysis, the higher M1 tortuosity observed in IADE-positive patients may point to a broader intracranial arterial phenotype. Previous work has linked MCA geometry to plaque distribution and hemodynamic conditions, suggesting that local arterial geometry may have vascular relevance [46]. Thus, one possible hypothesis is that MCA geometry may be related to deep cerebrovascular changes through local hemodynamic effects. However, the MCA analyses in our study were exploratory, and no validated threshold for abnormal MCA tortuosity is currently available. Multiple exploratory comparisons also increase the possibility of type I error. These observations are therefore hypothesis-generating and need confirmation in larger, independent cohorts.

Diabetes mellitus and previous stroke were less common in the IADE-positive group. A similar pattern was reported by Pico et al., who also found lower rates of diabetes and previous stroke among patients with IADE [1]. Together with the comparable carotid plaque burden and intima–media thickness between groups, these findings raise the possibility that IADE does not simply mirror conventional metabolic or atherosclerotic vascular disease. Hemodynamic, extracellular-matrix, or genetic susceptibility may also contribute, although this interpretation remains hypothesis-generating.

This study has several strengths. First, we systematically assessed IADE across both the anterior and posterior intracranial circulations rather than restricting the evaluation to the vertebrobasilar system. The morphology-based cICA classification was complemented by quantitative measurements of intracranial ICA tortuosity. Phenotype-specific sensitivity analyses and the exploratory MCA assessment provided additional information on the distribution of intracranial arterial tortuosity.

Several limitations should be acknowledged. The retrospective single-center design, modest sample size, and imaging-related exclusions may have introduced selection bias and limited generalizability. Some exclusions reflected unavailable source images from referring hospitals rather than clinical severity, although patients with severe ICH or clinical instability may still have been underrepresented. The macrovascular group and several IADE subtypes were small, resulting in limited precision and wide confidence intervals. Anterior-circulation IADE was defined primarily by morphology, an approach that is not a universally accepted diagnostic standard, and diameter-defined cICA ectasia was rare. The MCA analyses were exploratory and lacked validated tortuosity thresholds. The exact timing of vascular imaging was not systematically recorded, so acute ICH-related effects on vessel caliber or quantitative measurements cannot be excluded. Residual confounding from treatment-related factors, hypertension duration or severity, renal function, and other vascular comorbidities is also possible. Because the analysis was cross-sectional, temporal or causal relationships cannot be established, and longitudinal clinical outcomes were unavailable. Finally, this single-center cohort from Hunan Province may not generalize to other geographic or ethnic populations, and unmeasured genetic or environmental factors may have influenced the findings.

## 5. Conclusions

In conclusion, IADE was present in approximately one-third of patients with ICH and was observed more frequently in CSVD-related than in macrovascular ICH in this cohort. IADE was associated with deep CSVD imaging markers, whereas the MCA findings remain exploratory. Prospective multicenter studies with standardized vascular assessment and longitudinal follow-up are needed to determine the clinical and prognostic relevance of these associations.

## Figures and Tables

**Table 1:** Demographic and clinical characteristics of ICH patients with and without Intracranial Arterial Dolichoectasia (IADE).

Characteristic	Overall (*n* = 174)	IADE− (*n* = 122)	IADE+ (*n* = 52)	*p* Value
Age, years	57.2 ± 14.7	56.8 ± 15.4	58.3 ± 13.0	0.533
Male sex, *n* (%)	118 (67.8)	82 (67.2)	36 (69.2)	0.794
Hypertension, *n* (%)	140 (80.5)	96 (78.7)	44 (84.6)	0.367
Diabetes mellitus, *n* (%)	38 (21.8)	32 (26.2)	6 (11.5)	0.032
Dyslipidemia, *n* (%)	44 (25.3)	29 (23.8)	15 (28.8)	0.481
Coronary heart disease, *n* (%)	17 (9.8)	14 (11.5)	3 (5.8)	0.246
Current/former smoking, *n* (%)	67 (38.5)	46 (37.7)	21 (40.4)	0.739
Alcohol use, *n* (%)	49 (28.2)	38 (31.1)	11 (21.2)	0.180
Any previous stroke, *n* (%)	47 (27.0)	39 (32.0)	8 (15.4)	0.024
Previous ischemic stroke, *n* (%)	20 (11.5)	19 (15.6)	1 (1.9)	0.010
Previous ICH, *n* (%)	27 (15.5)	20 (16.4)	7 (13.5)	0.625
Systolic blood pressure, mmHg	142.2 ± 25.4	139.5 ± 25.0	148.6 ± 25.4	0.030
Diastolic blood pressure, mmHg	85.2 ± 15.6	83.7 ± 16.0	88.9 ± 14.3	0.045
Vascular imaging modality, *n* (%)				0.726
MRA	140 (80.5)	99 (81.1)	41 (78.8)	
CTA	34 (19.5)	23 (18.9)	11 (21.2)	
ICH location, *n* (%)				0.873
Lobar	57 (32.8)	41 (33.6)	16 (30.8)	
Deep/infratentorial	102 (58.6)	70 (57.4)	32 (61.5)	
Uncertain	15 (8.6)	11 (9.0)	4 (7.7)	
ICH etiologic category, *n* (%)				0.049
CSVD-related	146 (83.9)	98 (80.3)	48 (92.3)	
Macrovascular	28 (16.1)	24 (19.7)	4 (7.7)	
ICH etiology subtype, *n* (%)				0.098
Arteriolosclerosis	40 (23.0)	29 (23.8)	11 (21.2)	
CAA	27 (15.5)	21 (17.2)	6 (11.5)	
Mixed CSVD	75 (43.1)	45 (36.9)	30 (57.7)	
Other rare CSVD	4 (2.3)	3 (2.5)	1 (1.9)	
Macrovascular lesion	28 (16.1)	24 (19.7)	4 (7.7)	
NIHSS score, median (IQR)	5.0 (2.0–12.0)	4.5 (2.0–12.5)	5.0 (3.0–8.0)	0.692
mRS score, median (IQR)	3.0 (2.0–4.0)	3.0 (2.0–5.0)	3.0 (2.0–4.0)	0.736
Hematoma volume, mL, median (IQR)	6.1 (2.6–13.6)	5.1 (2.6–12.0)	9.9 (3.1–19.1)	0.226
Mean carotid IMT, mm	0.89 ± 0.17	0.88 ± 0.16	0.92 ± 0.19	0.396
Carotid plaque, *n*/N (%)	54/69 (78.3)	42/52 (80.8)	12/17 (70.6)	0.377

Data are presented as mean ± SD, median (interquartile range), or *n* (%). *p* values were calculated using Welch’s *t* test, Mann–Whitney U test, chi-square test, or Fisher’s exact test, as appropriate. Available-case analyses were used for variables with missing data. Hematoma volume was available in 149 patients, NIHSS and mRS scores in 153 patients, and carotid ultrasound data in 69 patients. IADE, intracranial arterial dolichoectasia; ICH, intracerebral hemorrhage; BP, blood pressure; IS, ischemic stroke; CSVD, cerebral small vessel disease; CAA, cerebral amyloid angiopathy; CTA, computed tomography angiography; MRA, magnetic resonance angiography; NIHSS, National Institutes of Health Stroke Scale; mRS, modified Rankin Scale; IMT, intima–media thickness.

**Table 2:** CSVD imaging markers according to IADE status among patients with CSVD-related ICH.

CSVD Imaging Marker	Overall (*n* = 146)	IADE− (*n* = 98)	IADE+ (*n* = 48)	*p* Value
**Lacunes**				
Lacunes ≥ 1, *n* (%)	105 (71.9)	72 (73.5)	33 (68.8)	0.551
Deep lacunes ≥ 1, *n* (%)	76 (52.1)	50 (51.0)	26 (54.2)	0.721
**Cerebral microbleeds**				
Any CMBs ≥ 1, *n* (%)	123 (84.2)	78 (79.6)	45 (93.8)	0.027
Lobar CMBs ≥ 1, *n* (%)	94 (64.4)	60 (61.2)	34 (70.8)	0.255
Deep CMBs ≥ 1, *n* (%)	107 (73.3)	66 (67.3)	41 (85.4)	0.020
Infratentorial CMBs ≥ 1, *n* (%)	80 (54.8)	49 (50.0)	31 (64.6)	0.096
Total CMB count, median (IQR)	7.0 (2.0–19.8)	7.0 (1.0–18.8)	8.0 (2.8–20.2)	0.384
Deep CMB count, median (IQR)	2.5 (0.0–8.0)	2.0 (0.0–7.8)	3.0 (1.0–10.0)	0.141
**EPVS**				
Severe BG-EPVS, grade ≥ 3, *n* (%)	43 (29.5)	22 (22.4)	21 (43.8)	0.008
Severe CSO-EPVS, grade ≥ 3, *n* (%)	54 (37.0)	34 (34.7)	20 (41.7)	0.412
BG-EPVS grade, median (IQR)	2.0 (1.0–3.0)	2.0 (1.0–2.0)	2.0 (1.0–3.0)	0.012
CSO-EPVS grade, median (IQR)	2.0 (1.0–3.0)	2.0 (1.0–3.0)	2.0 (2.0–3.0)	0.216
**White matter hyperintensities**				
Deep WMH grade ≥ 2, *n* (%)	86 (58.9)	51 (52.0)	35 (72.9)	0.016
Periventricular WMH grade ≥ 3, *n* (%)	40 (27.4)	26 (26.5)	14 (29.2)	0.737
Deep WMH grade, median (IQR)	2.0 (1.0–2.0)	2.0 (1.0–2.0)	2.0 (1.0–2.0)	0.098
Periventricular WMH grade, median (IQR)	2.0 (1.0–3.0)	2.0 (1.0–3.0)	2.0 (1.0–3.0)	0.356
**CSVD burden scores**				
Global CSVD score, median (IQR)	4.0 (2.0–5.0)	3.0 (2.0–5.0)	4.0 (3.0–5.0)	0.049
HTNA-CSVD score, median (IQR)	3.0 (2.0–4.0)	3.0 (2.0–4.0)	3.0 (2.0–4.0)	0.020
CAA-CSVD score, median (IQR)	2.0 (1.0–3.0)	2.0 (1.0–3.0)	2.0 (1.0–3.0)	0.115

Data are presented as *n* (%) or median (interquartile range). Analyses were restricted to patients with CSVD-related ICH. *p* values were calculated using Pearson’s chi-square test or Fisher’s exact test for categorical variables and the Mann–Whitney U test for ordinal or nonnormally distributed continuous variables. CMB, cerebral microbleed; EPVS, enlarged perivascular spaces; BG, basal ganglia; CSO, centrum semiovale; WMH, white matter hyperintensity; CSVD, cerebral small vessel disease; HTNA-CSVD, hypertensive arteriopathy-related CSVD; CAA-CSVD, cerebral amyloid angiopathy-related CSVD.

**Table 3:** Association Between IADE and Selected CSVD Imaging Markers Among Patients With CSVD-Related ICH.

	IADE (+) (*n* = 48)	IADE (−) (*n* = 98)	Unadjusted OR (95% CI)	*p* Value	Adjusted OR (95% CI)	*p* Value	FDR q Value
Lacunes ≥ 1	33 (68.8)	72 (73.5)	0.79 (0.37–1.69)	0.552	1.03 (0.46–2.28)	0.946	—
Deep lacunes ≥ 1	26 (54.2)	50 (51.0)	1.13 (0.57–2.27)	0.721	1.37 (0.66–2.85)	0.403	—
Any CMBs ≥ 1	45 (93.8)	78 (79.6)	3.85 (1.08–13.67)	0.037	5.70 (1.38–23.56)	0.016	—
Deep CMBs ≥ 1	41 (85.4)	66 (67.3)	2.84 (1.15–7.03)	0.024	3.42 (1.27–9.20)	0.015	0.015
Infratentorial CMBs ≥ 1	31 (64.6)	49 (50.0)	1.82 (0.89–3.72)	0.098	2.08 (0.95–4.54)	0.067	—
Lobar CMBs ≥ 1	34 (70.8)	60 (61.2)	1.54 (0.73–3.23)	0.255	1.59 (0.71–3.56)	0.263	—
BG-EPVS grade ≥ 2	35 (72.9)	55 (56.1)	2.10 (0.99–4.46)	0.052	3.00 (1.15–7.85)	0.025	—
Severe BG-EPVS, grade ≥ 3	21 (43.8)	22 (22.4)	2.69 (1.28–5.64)	0.009	4.24 (1.72–10.46)	0.002	0.004
CSO-EPVS grade ≥ 2	41 (85.4)	67 (68.4)	2.71 (1.09–6.72)	0.031	2.64 (1.01–6.89)	0.047	—
Severe CSO-EPVS, grade ≥ 3	20 (41.7)	34 (34.7)	1.34 (0.66–2.73)	0.413	1.63 (0.76–3.51)	0.213	—
Periventricular WMH grade ≥ 3	14 (29.2)	26 (26.5)	1.14 (0.53–2.46)	0.737	1.32 (0.57–3.07)	0.513	—
Deep WMH grade ≥ 2	35 (72.9)	51 (52.0)	2.48 (1.17–5.25)	0.018	3.71 (1.59–8.65)	0.002	0.004
Arteriolosclerosis	11 (22.9)	29 (29.6)	0.71 (0.32–1.58)	0.397	0.83 (0.34–2.01)	0.680	—
CAA	6 (12.5)	21 (21.4)	0.52 (0.20–1.40)	0.197	0.37 (0.11–1.26)	0.113	—
Mixed CSVD	30 (62.5)	45 (45.9)	1.96 (0.97–3.98)	0.061	2.03 (0.94–4.38)	0.071	
Lobar ICH	14 (29.2)	32 (32.7)	0.85 (0.40–1.80)	0.670	0.82 (0.36–1.86)	0.636	—
Deep/infratentorial ICH	31 (64.6)	58 (59.2)	1.26 (0.61–2.57)	0.530	1.40 (0.64–3.05)	0.402	—

Multivariable models were adjusted for age, sex, hypertension, diabetes mellitus, and any previous stroke. FDR q values are shown for the three prioritized deep CSVD outcomes: deep CMBs ≥ 1, severe BG-EPVS, and deep WMH grade ≥ 2. Other outcomes were considered exploratory. Odds ratios indicate the odds of each CSVD marker in IADE-positive patients compared with IADE-negative patients. IADE, intracranial arterial dolichoectasia; CSVD, cerebral small vessel disease; ICH, intracerebral hemorrhage; CMB, cerebral microbleed; EPVS, enlarged perivascular spaces; BG, basal ganglia; CSO, centrum semiovale; WMH, white matter hyperintensity; OR, odds ratio; CI, confidence interval; FDR, false discovery rate.

## Data Availability

The authors confirm that the data supporting the findings of this study are available within the article.

## Supplementary Materials

The following supporting information can be downloaded at: https://www.mdpi.com/article/10.3390/jcm15176881/s1.

## Abbreviations

CLAS-ICH, Causal Classification System for Intracerebral Hemorrhage Subtypes; IADE, intracranial arterial dolichoectasia.

## References

[1] Pico F., Labreuche J., Seilhean D., Duyckaerts C., Hauw J.J., Amarenco P. (2007). Association of small-vessel disease with dilatative arteriopathy of the brain: Neuropathologic evidence. Stroke.

[2] Gutierrez J., Sacco R.L., Wright C.B. (2011). Dolichoectasia-an evolving arterial disease. Nat. Rev. Neurol..

[3] Pico F., Labreuche J., Touboul P.J., Amarenco P., Investigators G. (2003). Intracranial arterial dolichoectasia and its relation with atherosclerosis and stroke subtype. Neurology.

[4] Pico F., Labreuche J., Amarenco P. (2015). Pathophysiology, presentation, prognosis, and management of intracranial arterial dolichoectasia. Lancet Neurol..

[5] Del Brutto V.J., Gutierrez J., Goryawala M.Z., Sacco R.L., Rundek T., Romano J.G. (2021). Prevalence and Clinical Correlates of Intracranial Dolichoectasia in Individuals with Ischemic Stroke. Stroke.

[6] Smoker W.R., Corbett J.J., Gentry L.R., Keyes W.D., Price M.J., McKusker S. (1986). High-resolution computed tomography of the basilar artery: 2. Vertebrobasilar dolichoectasia: Clinical-pathologic correlation and review. AJNR Am. J. Neuroradiol..

[7] Park J.M., Koo J.S., Kim B.K., Kwon O., Lee J.J., Kang K., Lee J.S., Lee J., Bae H.J. (2013). Vertebrobasilar dolichoectasia as a risk factor for cerebral microbleeds. Eur. J. Neurol..

[8] Cao Y., Huang M.Y., Mao C.H., Wang X., Xu Y.Y., Qian X.J., Ma C., Qiu W.Y., Zhu Y.C. (2023). Arteriolosclerosis differs from venular collagenosis in relation to cerebrovascular parenchymal damage: An autopsy-based study. Stroke Vasc. Neurol..

[9] Raposo N., Zanon Zotin M.C., Seiffge D.J., Li Q., Goeldlin M.B., Charidimou A., Shoamanesh A., Jäger H.R., Cordonnier C., Klijn C.J.M. (2022). A Causal Classification System for Intracerebral Hemorrhage Subtypes. Ann. Neurol..

[10] De Simone M., Ciaglia E., Santurro A., Choucha A., Messuti B., Fasolino S., De Feo R., Romano D.G., Guerra G., De Luca A. (2026). Chronic Subdural Hematoma Is Not Subdural: Anatomical, Biological, and Therapeutic Implications of a Misleading Definition. Brain Sci..

[11] Pico F., Labreuche J., Touboul P.J., Leys D., Amarenco P. (2005). Intracranial arterial dolichoectasia and small-vessel disease in stroke patients. Ann. Neurol..

[12] Zhang D.P., Yin S., Zhang H.L., Li D., Song B., Liang J.X. (2020). Association between Intracranial Arterial Dolichoectasia and Cerebral Small Vessel Disease and Its Underlying Mechanisms. J. Stroke.

[13] Thiankhaw K., Ozkan H., Ambler G., Werring D.J. (2023). Relationships between intracranial arterial dolichoectasia and small vessel disease in patients with ischaemic stroke: A systematic review and meta-analysis. J. Neurol..

[14] Han F., Clancy U., Arteaga-Reyes C., Thrippleton M.J., Valdes Hernandez M.D.C., Jaime Garcia D., Stringer M.S., Backhouse E., Chappell F.M., Cheng Y. (2026). Implications of Cranial Arterial Stenosis and Dolichoectasia for Cerebral Small-Vessel Disease Etiopathogenesis: Findings From a Prospective Mild Stroke Cohort. Circulation.

[15] Kothari R.U., Brott T., Broderick J.P., Barsan W.G., Sauerbeck L.R., Zuccarello M., Khoury J. (1996). The ABCs of measuring intracerebral hemorrhage volumes. Stroke.

[16] Duering M., Biessels G.J., Brodtmann A., Chen C., Cordonnier C., de Leeuw F.E., Debette S., Frayne R., Jouvent E., Rost N.S. (2023). Neuroimaging standards for research into small vessel disease-advances since 2013. Lancet Neurol..

[17] Woo D., Rosand J., Kidwell C., McCauley J.L., Osborne J., Brown M.W., West S.E., Rademacher E.W., Waddy S., Roberts J.N. (2013). The Ethnic/Racial Variations of Intracerebral Hemorrhage (ERICH) study protocol. Stroke.

[18] Fazekas F., Kleinert R., Offenbacher H., Schmidt R., Kleinert G., Payer F., Radner H., Lechner H. (1993). Pathologic correlates of incidental MRI white matter signal hyperintensities. Neurology.

[19] Doubal F.N., MacLullich A.M., Ferguson K.J., Dennis M.S., Wardlaw J.M. (2010). Enlarged perivascular spaces on MRI are a feature of cerebral small vessel disease. Stroke.

[20] Castello J.P., Pasi M., Abramson J.R., Rodriguez-Torres A., Marini S., Demel S., Gilkerson L., Kubiszewski P., Charidimou A., Kourkoulis C. (2021). Contribution of Racial and Ethnic Differences in Cerebral Small Vessel Disease Subtype and Burden to Risk of Cerebral Hemorrhage Recurrence. Neurology.

[21] Charidimou A., Boulouis G., Frosch M.P., Baron J.C., Pasi M., Albucher J.F., Banerjee G., Barbato C., Bonneville F., Brandner S. (2022). The Boston criteria version 2.0 for cerebral amyloid angiopathy: A multicentre, retrospective, MRI-neuropathology diagnostic accuracy study. Lancet Neurol..

[22] Wang L., Liu Q., Yue D., Liu J., Fu Y. (2024). Cerebral Amyloid Angiopathy: An Undeniable Small Vessel Disease. J. Stroke.

[23] Ciurică S., Lopez-Sublet M., Loeys B.L., Radhouani I., Natarajan N., Vikkula M., Maas A.H.E.M., Adlam D., Persu A. (2019). Arterial Tortuosity. Hypertension.

[24] Smoker W.R., Price M.J., Keyes W.D., Corbett J.J., Gentry L.R. (1986). High-resolution computed tomography of the basilar artery: 1. Normal size and position. AJNR Am. J. Neuroradiol..

[25] Cao Y., Zhang D.D., Mu J.Y., Liu Y.M., Gao F., Han F., Zhai F.F., Zhou L.X., Ni J., Yao M. (2022). Different Types of Circulatory Inflammatory Biomarkers Associated with Cerebral Arterial Atherosclerosis and Dolichoectasia. Cerebrovasc. Dis..

[26] Lin L.M., Colby G.P., Jiang B., Uwandu C., Huang J., Tamargo R.J., Coon A.L. (2015). Classification of cavernous internal carotid artery tortuosity: A predictor of procedural complexity in Pipeline embolization. J. Neurointerv Surg..

[27] Koge J., Tanaka K., Yoshimoto T., Shiozawa M., Kushi Y., Ohta T., Satow T., Kataoka H., Ihara M., Koga M. (2022). Internal Carotid Artery Tortuosity: Impact on Mechanical Thrombectomy. Stroke.

[28] Hoshino T., Sato S., Kushi K., Tanaka Y., Mochizuki T., Ishikawa T., Shima S., Ryu B., Inoue T., Okada Y. (2024). Tortuosity of middle cerebral artery M1 segment and outcomes after mechanical thrombectomy. Interv. Neuroradiol..

[29] Gutierrez J., Bagci A., Gardener H., Rundek T., Ekind M.S.V., Alperin N., Sacco R.L., Wright C.B. (2014). Dolichoectasia Diagnostic Methods in a Multi-Ethnic, Stroke-Free Cohort: Results from the Northern Manhattan Study. J. Neuroimaging.

[30] Thiankhaw K., Panteleienko L., Stewart C.R., Oliver R., Mallon D., Ambler G., Werring D.J. (2025). Association of Intracranial Dolichoectasia and Cerebral Small Vessel Disease in Patients with Intracerebral Hemorrhage. J. Am. Heart Assoc..

[31] Nakamura Y., Hirayama T., Ikeda K. (2012). Clinicoradiologic features of vertebrobasilar dolichoectasia in stroke patients. J. Stroke Cerebrovasc. Dis..

[32] Shapiro M., Becske T., Riina H.A., Raz E., Zumofen D., Nelson P.K. (2014). Non-saccular vertebrobasilar aneurysms and dolichoectasia: A systematic literature review. J. Neurointerv Surg..

[33] Del Brutto O.H., Mera R.M., Del Brutto V.J., Costa A.F., Zambrano M., Brorson J. (2017). Basilar Artery Dolichoectasia: Prevalence and Correlates with Markers of Cerebral Small Vessel Disease in Community-Dwelling Older Adults. J. Stroke Cerebrovasc. Dis..

[34] Cao S., Zhu X., Wu Q., Ni X., He J., Cui P., Ge T., Wang J., Xu W., Xia M. (2021). Basilar Artery Dolichosis Increases the Risk of Long-Term Recurrence in Patients with Pontine Infarction: A Prospective Cohort Study. Front. Neurol..

[35] Wei F., Diedrich K.T., Fullerton H.J., deVeber G., Wintermark M., Hodge J., Kirton A., Dowling M.M., Benedict S.L., Bernard T.J. (2016). Arterial Tortuosity: An Imaging Biomarker of Childhood Stroke Pathogenesis?. Stroke.

[36] Zhang D.P., Peng Y.F., Zhang H.L., Ma J.G., Zhao M., Yin S., Wei T.T. (2019). Basilar Artery Tortuosity Is Associated with White Matter Hyperintensities by TIMP-1. Front. Neurosci..

[37] Yu C., Li Y., Xiao Y., Li Q., Lu W., Qiu J., Wang F., Li J. (2024). Characterization of posterior circulation blood perfusion in patients with different degrees of basilar artery tortuosity. Neurol. Sci..

[38] Hong J.M., Chung C.S., Bang O.Y., Yong S.W., Joo I.S., Huh K. (2009). Vertebral artery dominance contributes to basilar artery curvature and peri-vertebrobasilar junctional infarcts. J. Neurol. Neurosurg. Psychiatry.

[39] Gutierrez J., DiTullio M., K Cheung Y.K., Alperin N., Bagci A., L Sacco R., B Wright C., Sv Elkind M., Rundek T. (2019). Brain arterial dilatation modifies the association between extracranial pulsatile hemodynamics and brain perivascular spaces: The Northern Manhattan Study. Hypertens. Res..

[40] Kearns K.N., Yagmurlu K., Chen C.J., Jane J., Park M.S., Kalani M.Y.S. (2019). Deletion of 6p25.3 Is Associated with Cerebrovascular Dolichoectasia: Report of 2 Cases. Pediatr. Neurosurg..

[41] Miyatake S., Schneeberger S., Koyama N., Yokochi K., Ohmura K., Shiina M., Mori H., Koshimizu E., Imagawa E., Uchiyama Y. (2018). Biallelic COLGALT1 variants are associated with cerebral small vessel disease. Ann. Neurol..

[42] Chen L., Li S., Xie F., Hu X., Lv W. (2025). Cerebral small vessel disease associated with COL4A1 and COL4A2 duplication: Clinical and MRI features resembling CADASIL. Neurol. Sci..

[43] Renard D., Mine M., Pipiras E., Labauge P., Delahaye A., Benzacken B., Tournier-Lasserve E. (2014). Cerebral small-vessel disease associated with COL4A1 and COL4A2 gene duplications. Neurology.

[44] Morales-Verdugo J., Perez-Rojas F., Figueroa-Figueroa A., Lagos-Fica J., Vera-Paredes J., Garcia-Suarez O., Cabezas-Salgado J., Orellana-Cortes F. (2025). Detection, cerebrovascular complications and risk factors associated with vertebrobasilar dolichoectasia: A scoping review. Front. Neurol..

[45] Menshawi K., Mohr J.P., Gutierrez J. (2015). A Functional Perspective on the Embryology and Anatomy of the Cerebral Blood Supply. J. Stroke.

[46] Yu Y.N., Li M.L., Xu Y.Y., Meng Y., Trieu H., Villablanca J.P., Gao S., Feng F., Liebeskind D.S., Xu W.H. (2018). Middle cerebral artery geometric features are associated with plaque distribution and stroke. Neurology.

