# Supplementary material for: Intracranial Arterial Dolichoectasia in Spontaneous Intracerebral Hemorrhage: A Retrospective Cross-Sectional Study of Morphometric Features and Cerebral Small Vessel Disease
Source: J Clin Med. 2026 Sep 5;15(17):6881. doi: 10.3390/jcm15176881 (PMC13566162; doi:10.3390/jcm15176881)
Supplement: Supplementary file 1 [file jcm-15-06881-s001.zip › jcm-4449276-supplementary.pdf]

Supplementary Table S1. Intracranial ICA length and tortuosity index across cavernous ICA morphological categories

Side	cICA morphology	n	Centerline length, mm Median (IQR)	Intracranial ICA-TI Median (IQR)
Right	Type I	117	66.1 (63.3–70.9)	175.4 (166.7–188.7)
	Type II	26	71.6 (69.0–75.6)	188.7 (179.4–199.0)
	Type III	27	80.9 (74.8–83.3)	217.4 (198.0–227.3)
	Type IV	3	83.3 (77.8–89.0)	212.8 (204.4–245.3)
Left	Type I	117	67.1 (63.8–70.8)	185.2 (169.5–200.0)
	Type II	35	71.5 (68.6–76.0)	196.1 (183.5–212.8)
	Type III	18	79.5 (74.7–84.5)	217.4 (209.4–226.0)
	Type IV	4	89.2 (85.5–90.2)	263.9 (250.0–279.8)

Table note. Data are presented as median (interquartile range). Intracranial ICA measurements extended from the petrous genu to the terminal ICA bifurcation. The tortuosity index was calculated as the centerline length divided by the straight-line distance and multiplied by 100. Overall comparisons across cavernous ICA Types I–IV were performed using the Kruskal–Wallis test. Significant differences were observed for centerline length and intracranial ICA-TI on both sides (all $P<0.001$). One right intracranial ICA had incomplete quantitative measurements and was excluded from the centerline length and TI analyses; its cavernous ICA morphology remained assessable. ICA, internal carotid artery; TI, tortuosity index; IQR, interquartile range.

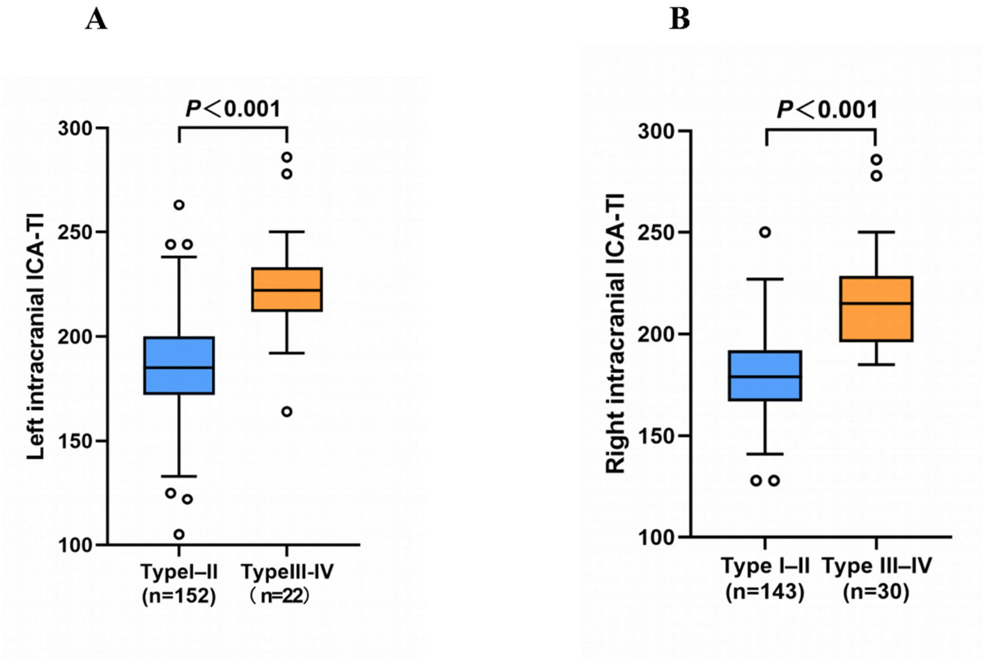

Supplementary Figure S1. Quantitative comparison of cavernous ICA morphological classification using the intracranial ICA tortuosity index.

Intracranial ICA-TI was compared between non-tortuous cavernous ICA morphology (Types I–II) and tortuous morphology (Types III–IV) for the left ICA (A) and right ICA (B). The tortuosity index was calculated as the centerline length divided by the straight-line distance and multiplied by 100. Boxes represent the median and interquartile range, and whiskers extend to 1.5 times the interquartile range. Outlying observations are shown as individual points. Between-group comparisons were performed using the Mann–Whitney U test. Intracranial ICA-TI was higher in Types III–IV than in Types I–II on both sides (both $P<0.001$). One right ICA was excluded because of incomplete quantitative measurements.

Supplementary Table S2. Distribution of Macrovascular ICH Etiologies According to IADE Status

Macrovascular etiology	n (% of macrovascular ICH)	IADE−, n (%)	IADE+, n (%)
Cerebral cavernous malformation	14 (50.0)	12 (85.7)	2 (14.3)
Arteriovenous malformation	9 (32.1)	9 (100.0)	0 (0.0)
Dural arteriovenous fistula	3 (10.7)	2 (66.7)	1 (33.3)
Moyamoya disease	2 (7.1)	1 (50.0)	1 (50.0)
Total	28 (100.0)	24 (85.7)	4 (14.3)

Table note. Data are presented as n (%). Analyses were restricted to patients with macrovascular ICH (n=28). Percentages in the IADE- and IADE+ columns were calculated within each etiologic subgroup. IADE, intracranial arterial dolichoectasia; ICH, intracerebral hemorrhage.

Supplementary Table S3. Phenotype-Specific Sensitivity Analyses of the Associations Between IADE and Deep CSVD Imaging Markers

IADE phenotype	Outcome	Phenotype (+)	Phenotype (−)	Unadjusted OR (95% CI)	*P*	Adjusted OR (95% CI)	Adjusted *P*	FDR q
cICA tortuosity criterion	Deep CMBs ≥1	29/34 (85.3%)	78/112 (69.6%)	2.53 (0.90–7.09)	0.078	2.98 (0.998–8.90)	0.0504	0.0504
	Severe BG-EPVS, grade ≥3	16/34 (47.1%)	27/112 (24.1%)	2.80 (1.26–6.23)	0.012	2.94 (1.20–7.24)	0.019	0.028
	Deep WMH grade ≥2	26/34 (76.5%)	60/112 (53.6%)	2.82 (1.17–6.76)	0.02	3.09 (1.23–7.78)	0.016	0.028
VBD criterion	Deep CMBs ≥1	15/19 (78.9%)	92/127 (72.4%)	1.43 (0.44–4.59)	0.552	1.27 (0.36–4.48)	0.714	0.714
	Severe BG-EPVS, grade ≥3	6/19 (31.6%)	37/127 (29.1%)	1.12 (0.40–3.18)	0.827	1.91 (0.58–6.30)	0.288	0.432
	Deep WMH grade ≥2	12/19 (63.2%)	74/127 (58.3%)	1.23 (0.45–3.33)	0.687	1.95 (0.65–5.83)	0.235	0.432
Combined IADE phenotype	Deep CMBs ≥1	41/48 (85.4%)	66/98 (67.3%)	2.84 (1.15–7.03)	0.024	3.42 (1.27–9.20)	0.015	0.015
	Severe BG-EPVS, grade ≥3	21/48 (43.8%)	22/98 (22.4%)	2.69 (1.28–5.64)	0.009	4.24 (1.72–10.46)	0.002	0.004
	Deep WMH grade ≥2	35/48 (72.9%)	51/98 (52.0%)	2.48 (1.17–5.25)	0.018	3.71 (1.59–8.65)	0.002	0.004

Table note. Data are presented as n/N (%). Analyses were restricted to patients with CSVD-related ICH (n=146). cICA tortuosity was defined as Type III–IV morphology on either side, and VBD according to the Smoker criteria. The combined IADE phenotype was defined as the presence of cICA tortuosity and/or VBD. Logistic regression models were adjusted for age, sex, hypertension, diabetes mellitus, and any previous stroke. Benjamini–Hochberg FDR correction was applied across the three prioritized deep CSVD outcomes within each phenotype analysis.

Supplementary Table S4. Subtype-Stratified Analysis of IADE and Deep CSVD Imaging Markers

Outcome	IADE subtype	n/N (%)	Unadjusted OR (95% CI)	*P* value	Adjusted OR (95% CI)	*P* value
**Deep CMBs ≥1**	**No IADE**	66/98 (67.3)	Reference	—	Reference	—
	**Isolated cICA tortuosity**	26/29 (89.7)	4.20 (1.18–14.93)	0.026	5.75 (1.47–22.51)	0.012
	**Isolated VBD**	12/14 (85.7)	2.91 (0.61–13.78)	0.178	3.45 (0.61–19.49)	0.161
	**Combined cICA–VBD**	3/5 (60.0)	0.73 (0.12–4.57)	0.734	0.60 (0.09–3.93)	0.591
**Severe BG-EPVS, grade ≥3**	**No IADE**	22/98 (22.4)	Reference	—	Reference	—
	**Isolated cICA tortuosity**	15/29 (51.7)	3.70 (1.55–8.83)	0.003	4.98 (1.79–13.91)	0.002
	**Isolated VBD**	5/14 (35.7)	1.92 (0.58–6.32)	0.284	5.81 (1.31–25.79)	0.021
	**Combined cICA–VBD**	1/5 (20.0)	0.86 (0.09–8.13)	0.898	0.90 (0.08–9.61)	0.929
**Deep WMH grade ≥2**	**No IADE**	51/98 (52.0)	Reference	—	Reference	—
	**Isolated cICA tortuosity**	23/29 (79.3)	3.53 (1.32–9.43)	0.012	4.49 (1.57–12.83)	0.005
	**Isolated VBD**	9/14 (64.3)	1.66 (0.52–5.31)	0.394	3.57 (0.96–13.33)	0.058
	**Combined cICA–VBD**	3/5 (60.0)	1.38 (0.22–8.64)	0.729	1.65 (0.24–11.47)	0.611

Table note. Data are presented as n/N (%). Analyses were restricted to patients with CSVD-related ICH (n=146). IADE subtypes were mutually exclusive: isolated cICA tortuosity, isolated VBD, combined cICA–VBD involvement, or no IADE. Odds ratios were estimated using the no-IADE group as the reference. Multivariable logistic regression models were adjusted for age, sex, hypertension, diabetes mellitus, and any previous stroke. Estimates for the combined cICA–VBD subgroup should be interpreted cautiously because of the small sample size (n=5).

Supplementary Table S5. Additional Covariate-Adjusted Sensitivity Analyses of the Associations Between IADE and Deep CSVD Imaging Markers

Outcome	Primary model, aOR (95% CI)	*P* value	FDR q	Sensitivity Model 1, aOR (95% CI)	*P* value	FDR q	Sensitivity Model 2, aOR (95% CI)	*P* value	FDR q
**Deep CMBs ≥1**	3.42 (1.27–9.20)	0.015	0.015	3.48 (1.29–9.38)	0.014	0.014	3.17 (1.16–8.64)	0.024	0.024
**Severe BG-EPVS, grade ≥3**	4.24 (1.72–10.46)	0.002	0.004	4.56 (1.81–11.46)	0.001	0.004	3.86 (1.56–9.56)	0.004	0.008
**Deep WMH grade ≥2**	3.71 (1.59–8.65)	0.002	0.004	3.71 (1.59–8.65)	0.002	0.004	3.37 (1.43–7.94)	0.005	0.008

Table note: Analyses were restricted to patients with CSVD-related ICH (n=146). The primary model was adjusted for age, sex, hypertension, diabetes mellitus, and any previous stroke. Sensitivity Model 1 additionally adjusted for vascular imaging modality (CTA vs. TOF-MRA; 27 and 119 patients, respectively). Sensitivity Model 2 additionally adjusted for current/former smoking, dyslipidemia, and alcohol use. Benjamini–Hochberg FDR correction was applied across the three prioritized deep CSVD outcomes within each model. No observations were excluded because of missing data.

Supplementary Table S6. MCA tortuosity index across morphology categories

Side	Morphology	n	MCA-TI, median (IQR)	Kruskal–Wallis P	Pairwise adjusted P
Left MCA	Straight	20	103.1(102.0–106.4)	<0.001	Straight vs. single-curve <0.001
Left MCA	Single-curve	110	111.1(106.4–117.6)		Straight vs. S-shaped <0.001
Left MCA	S-shaped	43	113.6(108.1–119.8)		Single-curve vs. S-shaped = 0.300
Right MCA	Straight	22	104.2(102.0–105.0)	<0.001	Straight vs. single-curve <0.001
Right MCA	Single-curve	106	109.3(105.5–116.3)		Straight vs. S-shaped<0.001;
Right MCA	S-shaped	46	112.4(108.7–124.6)		Single-curve vs. S-shaped = 0.023

Table note. TI values are presented as median and interquartile range. Morphology categories were coded as I = straight, C = single-curve, and S = S-shaped. Overall comparisons were performed using the Kruskal–Wallis test. Pairwise comparisons were performed using Dunn–Bonferroni post hoc tests. Left MCA morphology and TI were unavailable in one participant. For this participant, the available right MCA value and morphology were used to derive the patient-level MCA summary measures; TI, tortuosity index; IQR, interquartile range.

Supplementary Table S7. Association between IADE and MCA morphological markers in the overall cohort

MCA marker	IADE (-)	IADE (+)	Crude P	Adjusted β or OR	95% CI	Adjusted P
Maximum bilateral MCA-TI, median (IQR)	112.4 (107.5–120.1)	117.6 (109.6–130.3)	0.023	β = 6.91	1.06–12.76	0.021
Mean bilateral MCA-TI, median (IQR)	109.9 (105.9–115.5)	113.0 (107.0–122.4)	0.012	β = 5.29	1.42–9.15	0.008
Any curved MCA, n (%)	115 (94.3)	50 (96.2)	0.726	OR = 1.68	0.32–8.77	0.536
Highest-grade MCA morphology: S-shaped, n (%)	41 (33.6)	26 (50.0)	0.061	OR = 2.26	1.11–4.60	0.024

Table note. Continuous variables are presented as median (IQR), and categorical variables as n (%). Crude P values for MCA-TI were calculated using the Mann–Whitney U test. Crude P values for binary morphology markers were calculated using Fisher's exact test. Adjusted β values are unstandardized coefficients from multivariable linear regression models. Adjusted ORs were obtained from multivariable logistic regression models. All models were adjusted for age, sex, hypertension, diabetes mellitus, and any previous stroke. IADE− was the reference group. "Any curved MCA" was defined as a single-curve or S-shaped morphology on either side. "S-shaped highest-grade bilateral MCA morphology" was analyzed as a binary outcome against straight or single-curve morphology. These analyses were exploratory. IADE, intracranial arterial dolichoectasia; MCA, middle cerebral artery; TI, tortuosity index; IQR, interquartile range; OR, odds ratio; CI, confidence interval.

Supplementary Table S8. Sensitivity analysis of the association between MCA markers and IADE in the CSVD-related ICH subgroup

MCA marker	Overall cohort, adjusted OR (95% CI)	Overall P	CSVD-related ICH subgroup, adjusted OR (95% CI)	Subgroup P
Maximum bilateral MCA-TI, per 1-unit increase	1.022 (1.000–1.043)	0.047	1.017 (0.996–1.038)	0.114
Mean bilateral MCA-TI, per 1-unit increase	1.036 (1.005–1.069)	0.023	1.028 (0.998–1.060)	0.068
Any curved MCA	1.74 (0.32–9.54)	0.524	1.24 (0.20–7.68)	0.817
Highest-grade MCA morphology: S-shaped	2.37 (1.15–4.85)	0.019	2.32 (1.08–5.00)	0.032

Table note. IADE status was the dependent variable in all logistic regression models. Adjusted ORs were calculated after adjustment for age, sex, hypertension, diabetes mellitus, and any previous stroke. The overall cohort included all 174 eligible patients, whereas the subgroup analysis was restricted to the 146 patients with CSVD-related ICH. For continuous MCA-TI variables, ORs are reported per 1-unit increase. “Any curved MCA” was defined as a single-curve or S-shaped morphology on either side. “S-shaped highest-grade bilateral MCA morphology” was analyzed as a binary outcome against straight or single-curve morphology. These sensitivity analyses were exploratory. For the participant with unavailable left MCA measurements, patient-level MCA summary variables were derived from the available right MCA; no imputation of the missing left-sided measurement was performed. IADE, intracranial arterial dolichoectasia; CSVD, cerebral small vessel disease; ICH, intracerebral hemorrhage; MCA, middle cerebral artery; TI, tortuosity index; OR, odds ratio; CI, confidence interval.
